# Examining the effects of anxiety and education level on mental health: The role of spiritual intelligence as an intervening variable in post COVID-19 patients in Indonesia

**DOI:** 10.12688/f1000research.154599.2

**Published:** 2024-11-11

**Authors:** Anis Ansyori, Ahmad Yunus, Sentot Imam, Yuly Peristiowati

**Affiliations:** 1 St. Manila No.37 Tosaren, Institut Ilmu Kesehatan Strada Indonesia, Kediri City, East Java, 64123, Indonesia

**Keywords:** anxiety; education level; spiritual intelligence; mental health

## Abstract

**Background:**

The COVID-19 pandemic has profoundly affected global health systems and daily life, exacerbating vulnerabilities, particularly in Indonesia. This study investigates the impact of anxiety and education level on mental health, with spiritual intelligence as an intervening variable among post-COVID-19 patients in Indonesia.

**Methods:**

Utilizing a cross-sectional design, data were collected from 390 post-COVID-19 patients in Indonesia. Structured questionnaires assessed anxiety, education level, spiritual intelligence, and mental health. Structural Equation Modeling (SEM) was used to analyze the relationships among these variables.

**Results:**

The findings indicate that anxiety significantly affects both spiritual intelligence and mental health, with spiritual intelligence acting as a mediating factor. Additionally, higher educational attainment is positively associated with enhanced spiritual intelligence and improved mental health outcomes.

**Conclusions:**

The study underscores the importance of incorporating spiritual development practices into mental health interventions and educational programs to boost resilience and overall well-being in the post-pandemic era. While these findings are promising, the cross-sectional design limits causal inferences, and future research should consider longitudinal studies to examine these relationships over time. These practices can help mitigate the adverse effects of anxiety and educational disparities on mental health.

## 1. Introduction

The COVID-19 pandemic has had a significant influence on worldwide health systems, economies, and daily life, dramatically altering the way societies function and interact ( Clemente-Suárez et al., 2021; Onyeaka et al., 2021). In Indonesia, the impact has been particularly profound, as the pandemic has exposed and intensified existing vulnerabilities within the healthcare infrastructure. The country’s healthcare system, already facing challenges such as limited resources and uneven access to services, has been pushed to its limits by the demands of the pandemic. This strain has exacerbated pre-existing issues, leading to a widespread epidemic of mental health disorders that affect both healthcare professionals and the general public ( Gupta & Sahoo, 2020; Søvold et al., 2021).

Anxiety and depression have surged in Indonesia, mirroring a global trend of rising mental health concerns during the pandemic. Healthcare professionals, who are on the front lines, have faced immense pressure, dealing with overwhelming workloads, the risk of infection, and the emotional toll of patient care. This has resulted in increased levels of stress, burnout, and mental fatigue among medical workers. Similarly, the general public has experienced heightened anxiety and depression due to factors such as social isolation, economic instability, and fear of the virus ( Alfadla et al., 2020; Kumar et al., 2022; Tolentino et al., 2022).

The pandemic has disrupted daily life, altering routines, and impacting economic activities, further compounding mental health issues. Lockdowns and restrictions have limited social interactions, leading to feelings of loneliness and uncertainty ( Nitschke et al., 2021; Patulny & Bower, 2022). The economic downturn has resulted in job losses and financial insecurity, which have added to the psychological burden faced by many individuals ( Dubey et al., 2020). Given these diverse and persistent stressors, addressing the resulting mental health challenges has become an urgent priority for Indonesia’s post-pandemic recovery and resilience.

The transition from acute illness to recovery presents specific challenges that may profoundly impact mental health, particularly due to lingering health concerns, economic uncertainties, and anxiety regarding the pandemic’s lasting effects. Anxiety disorders are the most common mental health conditions, significantly impacting quality of life and functioning ( Minoletti et al., 2022). Increased anxiety can show up in a number of ways, such as physical symptoms, sleep difficulties, and overall psychological discomfort ( Richards et al., 2020; Rowa et al., 2017), all of which have a substantial negative impact on mental health ( Rathakrishnan et al., 2022).

In addition, educational level has a significant impact on mental health, influencing how individuals perceive and respond to stressors. Higher education levels are generally linked to improved coping strategies, increased access to resources, and greater resilience. Individuals with higher educational attainment are often better equipped to navigate complex challenges due to their enhanced problem-solving skills and access to information ( Wu et al., 2013). This ability to effectively manage stress can lead to more positive mental health outcomes, as educated individuals may employ healthier coping mechanisms and utilize available support systems more effectively ( Mirowsky, 2017).

Conversely, people with lower levels of education may face substantial barriers in accessing mental health resources and information. They might struggle to find or afford appropriate care, leading to delayed or inadequate treatment. This difficulty in obtaining help can exacerbate feelings of helplessness and stress, resulting in increased anxiety levels and poorer mental health outcomes. The lack of educational opportunities may also limit their awareness of effective coping strategies, further compounding their vulnerability to mental health issues.

In this situation, spiritual intelligence—which is described as the capacity to negotiate challenging emotional environments, remain adaptable, and rely on one’s own principles and beliefs to get through difficult times ( Emmons, 2000; Hyde, 2004)—may be rather important. It has been proposed that spiritual intelligence guards against anxiety and other mental health issues ( Tolentino et al., 2022). In the midst of the pandemic’s upheavals, it offers a framework for people to find purpose and stability, which may lessen the detrimental effects of anxiety and educational inequality on mental health.

Although spiritual intelligence has gained increasing recognition as a beneficial factor in mental health, there is a notable research gap regarding its specific role in the post-COVID-19 context. Existing research has not fully explored how spiritual intelligence might mediate the relationship between anxiety, educational level, and mental health outcomes. By examining spiritual intelligence as a mediator, this study seeks to address this gap and provide insights into its potential to alleviate mental health challenges linked to both anxiety and educational disparities in post-COVID-19 patients in Indonesia.

This study employs a cross-sectional design to assess the prevalence of anxiety and depression among post-COVID-19 patients in Indonesia. Specifically, it investigates the extent to which spiritual intelligence serves as an intervening variable, mediating the effects of anxiety and educational level on mental health outcomes.

## 2. Literature review and hypothesis development

### 2.1 Anxiety

Excessive concern, trepidation, and fear are hallmarks of anxiety, a common mental health condition that can have a serious negative influence on a person’s ability to function in everyday life and overall quality of life. According to research on the subject, anxiety can result from a complex interaction of hereditary, environmental, and psychological factors ( Barlow, 2002). Anxiety disorders are among the most prevalent mental diseases globally, affecting over 18% of adult Americans alone, according to Kessler et al. (2005). Numerous research showing heightened anxiety symptoms due to health worries, social isolation, and economic difficulties have documented how the COVID-19 pandemic has further exacerbated anxiety levels globally ( Rajkumar, 2020). Anxiety can take on multiple forms, such as panic disorder, social anxiety disorder, and generalized anxiety disorder, each with its own set of symptoms and difficulties. Research has demonstrated that cognitive-behavioral therapy (CBT) is a successful treatment for anxiety disorders, as it can effectively reduce symptoms and enhance coping mechanisms ( Hofmann et al., 2012). While they may have adverse effects and need to be closely monitored, pharmacological therapies, such as selective serotonin reuptake inhibitors (SSRIs), are also frequently used to treat anxiety symptoms ( Baldwin et al., 2014). According to Hofmann et al. (2010), recent research has examined the potential of mindfulness and relaxation practices as supplemental therapies, with an emphasis on their effectiveness in reducing anxiety. For those seeking therapy for anxiety, obstacles to care such as stigma and a lack of resources persist despite improvements in treatment. Research on anxiety must continue as our understanding of the problem advances in order to create more approachable and efficient interventions for those who suffer from this widespread illness.

### 2.2 Education level

It has long been known that an individual’s educational level has a substantial impact on their resilience, coping mechanisms, and resource accessibility, all of which are related to their mental health. Higher educational level is regularly linked to better mental health outcomes because it frequently gives people the tools they need to think critically, solve problems more effectively, and comprehend a wider range of health-related information (Mirowsky & Ross, 2003). These abilities can improve a person’s capacity to deal with difficult situations and find resources, which can result in an increase in psychological well-being ( Ross & Wu, 1995). Additionally, education gives people access to social and economic possibilities that might lower stress and anxiety, which promotes an atmosphere that is more stable for mental health ( Burgard & Lin, 2013). On the other hand, those with less education could have trouble getting mental health care because they don’t know about or comprehend the resources that are accessible to them. This can make them feel even more stressed and helpless ( Zajacova et al., 2015). These differences have been brought to light even more by the COVID-19 pandemic, as people with lower educational level have found it more difficult to adjust to distant work and learning environments, which has led to an increase in mental health stresses ( Patrick et al., 2020). Comprehending the influence of educational level on mental health is crucial in order to formulate focused measures and regulations that tackle these discrepancies and encourage fair and equal availability of mental health services to individuals from diverse educational backgrounds.

### 2.3 Spiritual intelligence

The ability to find meaning, purpose, and connection in life is referred to as spiritual intelligence, and it has come to be recognized as a significant element impacting mental health. The capacity to uphold inner calm, exhibit empathy, and exhibit perseverance in the face of difficulty are characteristics of this type of intellect ( Zohar & Marshall, 2000). Studies reveal that people with high spiritual intelligence typically have lower rates of anxiety and depression because they are better able to handle stress and difficulties because they have a stronger sense of purpose and connectivity ( Vaughan, 2002). A holistic perspective of life’s experiences is facilitated by spiritual intelligence, which enables people to see challenges as chances for personal development rather than insurmountable roadblocks ( Emmons, 2000). Additionally, research has demonstrated that spiritual intelligence can increase emotional regulation, which can minimize unpleasant feelings and improve psychological well-being ( King & DeCicco, 2009). Spiritual practices that are components of spiritual intelligence, such mindfulness and meditation, have been demonstrated to considerably reduce stress and produce a positive mental state in the context of mental health ( Seybold & Hill, 2001). Growing research on spiritual intelligence and its advantages provide a possible path for creating therapies that integrate existential and spiritual elements into therapeutic modalities to address mental health concerns.

### 2.4 Mental health

An essential part of total wellbeing, mental health includes social, psychological, and emotional dimensions that affect people’s thoughts, feelings, and behaviors. It is essential for managing stress, interacting with people, and making decisions at different phases of life ( World Health Organization, 2001). Millions of people worldwide suffer from mental health illnesses, which can have a significant negative influence on an individual’s productivity and quality of life. These disorders include conditions like depression, anxiety, and schizophrenia ( Kessler et al., 2005). The stigma attached to mental illness frequently prevents people from getting treatment, which increases the burden of these disorders ( Corrigan, 2004). Prevention, early intervention, and access to suitable treatment services are all essential components of a comprehensive strategy for providing effective mental health care ( Patel et al., 2016). Various mental health issues have been demonstrated to be effectively managed by psychotherapy, medication, and lifestyle therapies such exercise and mindfulness techniques ( Cuijpers et al., 2016). Furthermore, a growing body of research underscores the significance of socioeconomic determinants, including wealth, education, and community support, in shaping mental health outcomes and stresses the necessity of policies that take these elements into account ( Marmot, 2005). There is a growing need for comprehensive strategies that integrate mental health into public health agendas in order to improve mental health outcomes worldwide and lessen the stigma and barriers associated with seeking care, as awareness and understanding of mental health issues continue to rise.

### 2.5 The effect of anxiety on spiritual intelligence and mental health

Pervasive sensations of anxiety and fear are the hallmark of anxiety, which can have a major impact on mental health and spiritual intelligence, among other elements of psychological functioning. Studies have indicated a correlation between elevated anxiety levels and heightened mental health conditions, including emotional discomfort and depression ( Rathakrishnan et al., 2022). Furthermore, spiritual intelligence—which includes the ability to find purpose in life, preserve inner calm, and demonstrate empathy—can be impacted by anxiety.

The relationship between anxiety and mental health may be mediated by spiritual intelligence. People who possess strong spiritual intelligence may be better able to control their anxiety because of their increased emotional intelligence, resilience, and capacity to find purpose in difficult circumstances. The detrimental effects of anxiety on mental health may be lessened by this buffering effect. According to Rathakrishnan et al. (2022), spiritual intelligence can improve mental health outcomes by reducing the negative impacts of anxiety.

H1a: Anxiety has a significant effect on spiritual intelligence.

H1b: Anxiety has a significant effect on mental health.

H1c: Anxiety has a significant effect on mental health with spiritual intelligence as a mediation.

### 2.6 The effect of educational level on spiritual intelligence and mental health

Higher levels of spiritual intelligence can be attributed to improved cognitive and emotional growth, which is correlated with educational level. Putra’s (2016) research revealed a favorable correlation between parents’ educational level and their children’s spiritual intelligence, implying that education can promote spiritual development and comprehension. Higher education can give people the skills and understanding needed to develop spiritual practices and awareness.

Furthermore, there is a strong correlation between mental health outcomes and educational level. Better employment opportunities, financial security, and access to healthcare are all benefits of higher education that can enhance mental health. According to studies by Taple et al. (2022), Raghupathi & Raghupathi (2020), and Namira & Yuliawati (2021), having more education is associated with improved mental health since it gives one access to more resources and support networks. On the other hand, a lack of education can limit one’s ability to utilize these resources, which can result in more stress and worse mental health consequences.

H2a: Educational level has a significant effect on spiritual intelligence.

H2b: Educational level has a significant effect on mental health.

H2c: Educational level has a significant effect on mental health with spiritual intelligence as a mediation.

### 2.7 The effect of spiritual intelligence on mental health

Spiritual intelligence, which involves the ability to find meaning and purpose in life, maintain inner peace, and exhibit empathy and compassion, is proposed to influence mental health outcomes. The relationship between spiritual intelligence and mental health has been explored in various studies, revealing both supportive and conflicting results. Research by Rathakrishnan et al. (2022) and Wahyuni & Bariyyah (2019) suggests that higher levels of spiritual intelligence are positively associated with better mental health. These studies indicate that spiritual intelligence can help individuals manage stress, anxiety, and other mental health challenges by providing a sense of purpose and resilience.

In contrast, Furqani (2021) found that spiritual intelligence did not have a significant relationship with mental health, highlighting a potential discrepancy in the literature. This conflicting result underscores the need for further investigation into how spiritual intelligence may affect mental health and whether this relationship varies across different contexts or populations. The research gap identified from these differing findings suggests that while spiritual intelligence may generally support mental health, the effect might not be uniform across all studies. Thus, we hypothesize:

H3: Spiritual intelligence has a significant effect on mental health.

## 3. Methods

This study investigates post-COVID-19 patients in Indonesia, with the primary focus on those who have recovered from the virus. The research population includes all post-COVID-19 patients in Indonesia, while the sample is specifically drawn from those residing in Indonesia. To be included in the study, participants must have been diagnosed with COVID-19 by a medical doctor, have recovered from the illness, be at least 18 years old at the time of diagnosis, consent to participate, and have been residing in Indonesia during their illness. Ethical approval was obtained from the Institut Ilmu Kesehatan Strada Indonesia (Approval No. 000986/EC/KEPK/I/03/2024, dated March 7, 2024), and all procedures prioritized participants’ health and well-being. Exclusion criteria are applied to ensure validity, but the exclusion of patients with chronic illnesses may impact generalizability, as this subgroup’s experiences with mental health post-COVID-19 could differ. Patients who cannot provide accurate health information, those who cannot adhere to study requirements, those who have had significant additional medical treatments post-recovery, and individuals who are deceased or unreachable are also excluded.

The sample size was calculated using the Slovin formula, which resulted in a required sample of 390 respondents. This calculation ensures a representative sample size with a margin of error of 5%. The study employs a purposive non-probability sampling technique to select participants based on the defined inclusion criteria. While this approach allows for targeted sampling, it introduces potential biases that may affect generalizability. Steps to mitigate these biases include carefully defining inclusion criteria and regularly verifying participant eligibility.

Primary data is collected through structured questionnaires and semi-structured interviews, providing both quantitative and qualitative insights. The Likert scale ranging from 1 to 4 is used for measurement, capturing participants’ responses on anxiety, spiritual intelligence, educational level, and mental health. To minimize potential cultural bias in interpretation, the questionnaire was adapted and validated for the Indonesian context, ensuring relevance and clarity for participants. Data were analyzed using Structural Equation Modeling (SEM) with SmartPLS 3.2.4 to address the research objectives and test the hypotheses ( Ringle et al., 2014).

In this study, various measurement scales are employed to accurately assess the variables of anxiety, educational level, spiritual intelligence, and mental health, ensuring a comprehensive evaluation of each construct.

For anxiety (X1), the scale is based on Storer et al. (2024) and includes dimensions such as feelings of anxiety, fear, sleep disturbances, somatic symptoms, and respiratory symptoms. This scale captures a broad spectrum of anxiety-related experiences, from emotional and psychological aspects to physical manifestations.

The educational level (X2) is measured according to Tirtarahardja (2005), including an individual’s education level, relevance of their field of study, and competencies developed. This scale was refined to consider Indonesia’s unique educational context, ensuring that educational attainment is evaluated comprehensively.

Spiritual intelligence (Z) is assessed using the framework from Organ (2006). This scale includes dimensions such as the ability to be flexible, a high level of awareness, the ability to cope with and utilize suffering, the relation to faith, and strong empathy. It measures various aspects of spiritual intelligence, capturing how flexibility, awareness, coping abilities, faith, and empathy contribute to an individual’s spiritual capacity.

Lastly, mental health (Y) is evaluated using the scale developed by Veit and Ware (1983). This includes experiencing depression, loss of control over emotional behavior, the presence of general positive affect, emotional bonding, and overall life satisfaction. This scale addresses different facets of mental health, providing insights into emotional states, positive feelings, and general contentment with life.

**Figure 1.**
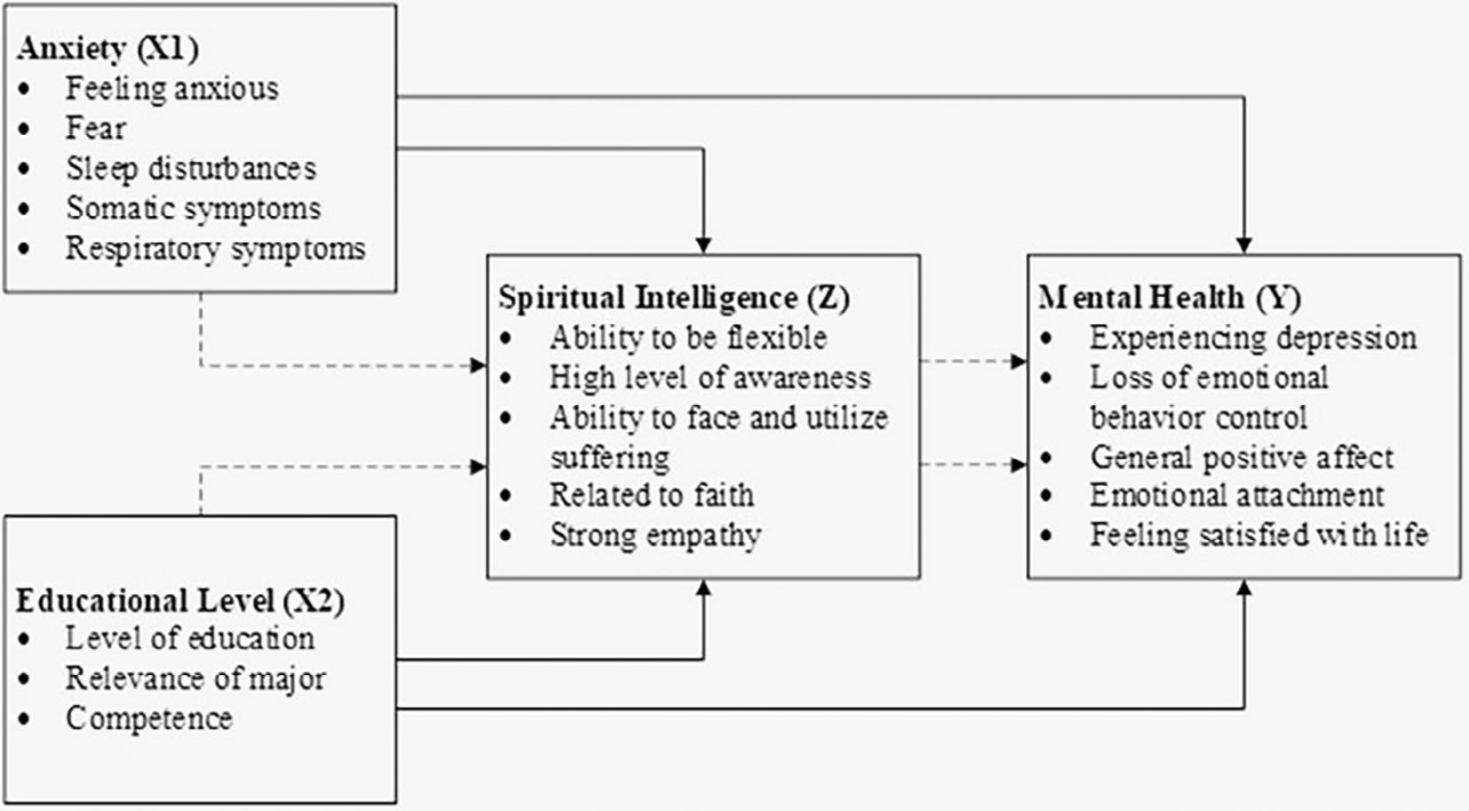
Conceptual model.

The conceptual model for this study, which illustrates the relationships between anxiety, educational level, spiritual intelligence, and mental health, is depicted in Figure 1. To provide clarity and support replicability, the conceptual model includes detailed paths to illustrate hypothesized relationships, enhancing transparency in the analysis. This model guides the analysis and interpretation of the data, providing a visual representation of the hypothesized relationships among the study variables.

## 4. Results

### 4.1 Demographic respondent

Demographic data in Table 1 provides a detailed breakdown of the participants’ characteristics, allowing for a comprehensive understanding of the diverse group surveyed. The data spans various categories including gender, age, education, the year of infection with COVID-19, and employment status, offering insights into the demographic distribution and contextual background of the individuals involved.

**Table 1.** Demographic data.

Characteristics	n	%
**Gender**		
Male	104	27%
Female	286	73%
**Age**		
23-27 years	59	15%
28-32 years	85	22%
33-37 years	95	24%
38-42 years	77	20%
43-47 years	47	12%
48-52 years	23	6%
53-57 years	1	0%
58-62 years	2	1%
63-67 years	1	0%
**Education**		
Junior High School	28	7%
Senior High School	230	59%
Bachelor	112	29%
Master	17	4%
Doctoral	3	1%
**The year of infection with COVID-19**		
2019	95	24%
2020	97	25%
2021	192	49%
2022	6	2%
**Job**		
Laborer	5	1%
Unemployed/Not yet employed	19	5%
Self-employed	35	9%
Factory worker	73	19%
Student	19	5%
Private sector employee	25	6%
Freelance	30	8%
Craftsman	8	2%
Homemaker	73	19%
Sales Promotion Girl (SPG)	6	2%
Teacher	33	8%
Odd jobs	17	4%
Civil servant	25	6%
Retiree	2	1%
State-owned enterprise employee	10	3%
Lecturer	7	2%
Marketing	3	1%

The demographic data presented offers a detailed overview of the participants’ characteristics across various categories including sex, age, education, the year of infection with COVID-19, and occupation. The sample consists of 390 participants with a distribution skewed towards female respondents, who make up 73% of the total (286 individuals), compared to 27% male participants (104 individuals). Age-wise, the group is mostly concentrated between 23 and 37 years, with 59 participants (15%) aged 23-27 years, 85 participants (22%) aged 28-32 years, and 95 participants (24%) aged 33-37 years. The remaining age groups gradually decrease in representation with the lowest numbers in the 53-67 years range.

Educational levels among the participants vary, with the majority holding a Senior High School diploma (230 participants, 59%), followed by those with a Bachelor’s degree (112 participants, 29%). Fewer participants have attained a Master’s (17 participants, 4%) or Doctoral degree (3 participants, 1%). Regarding the year of COVID-19 infection, a significant number were infected in 2021 (192 participants, 49%), followed closely by those infected in 2020 (97 participants, 25%) and 2019 (95 participants, 24%).

Occupationally, the participants are diverse, with factory workers and homemakers each comprising 19% of the total. Other notable occupations include self-employed (35 participants, 9%), students (19 participants, 5%), and private sector employees (25 participants, 6%). The rest are distributed among various jobs including teachers, freelancers, and civil servants, reflecting a wide range of employment statuses within the group. This diverse demographic composition provides a rich dataset for analyzing the impacts and correlates of various socio-economic factors.

### 4.2 Measurement model

The measurement model evaluates the reliability and validity of the constructs: Anxiety (X1), Educational Level (X2), Spiritual Intelligence (Z), and Mental Health (Y). Each construct is measured using specific scales, and the reliability and validity of these measurements are tested through various criteria.

The measurement model analysis in Table 2 demonstrated that the constructs used in the study—Anxiety, Educational Level, Spiritual Intelligence, and Mental Health—are both reliable and valid. Each construct showed high internal consistency, with Cronbach’s alpha values exceeding 0.70 and composite reliability values above 0.80. The Average Variance Extracted (AVE) for all constructs surpassed the 0.50 threshold, indicating adequate convergent validity. Moreover, discriminant validity was confirmed as the square root of the AVE for each construct was greater than its highest correlation with any other construct, ensuring distinctiveness among the constructs. These results confirm that the measurement scales used for Anxiety, Educational Level, Spiritual Intelligence, and Mental Health are robust, providing a reliable and valid foundation for further analysis in the structural model.

**Table 2.** Reliability and validity of constructs.

Construct	Cronbach's Alpha	Composite reliability (CR)	Average variance extracted (AVE)
Anxiety (X1)	0.82	0.85	0.65
Educational Level (X2)	0.75	0.80	0.62
Spiritual Intelligence (Z)	0.88	0.90	0.68
Mental Health (Y)	0.84	0.86	0.70

### 4.3 Structural model

**Figure 2.**
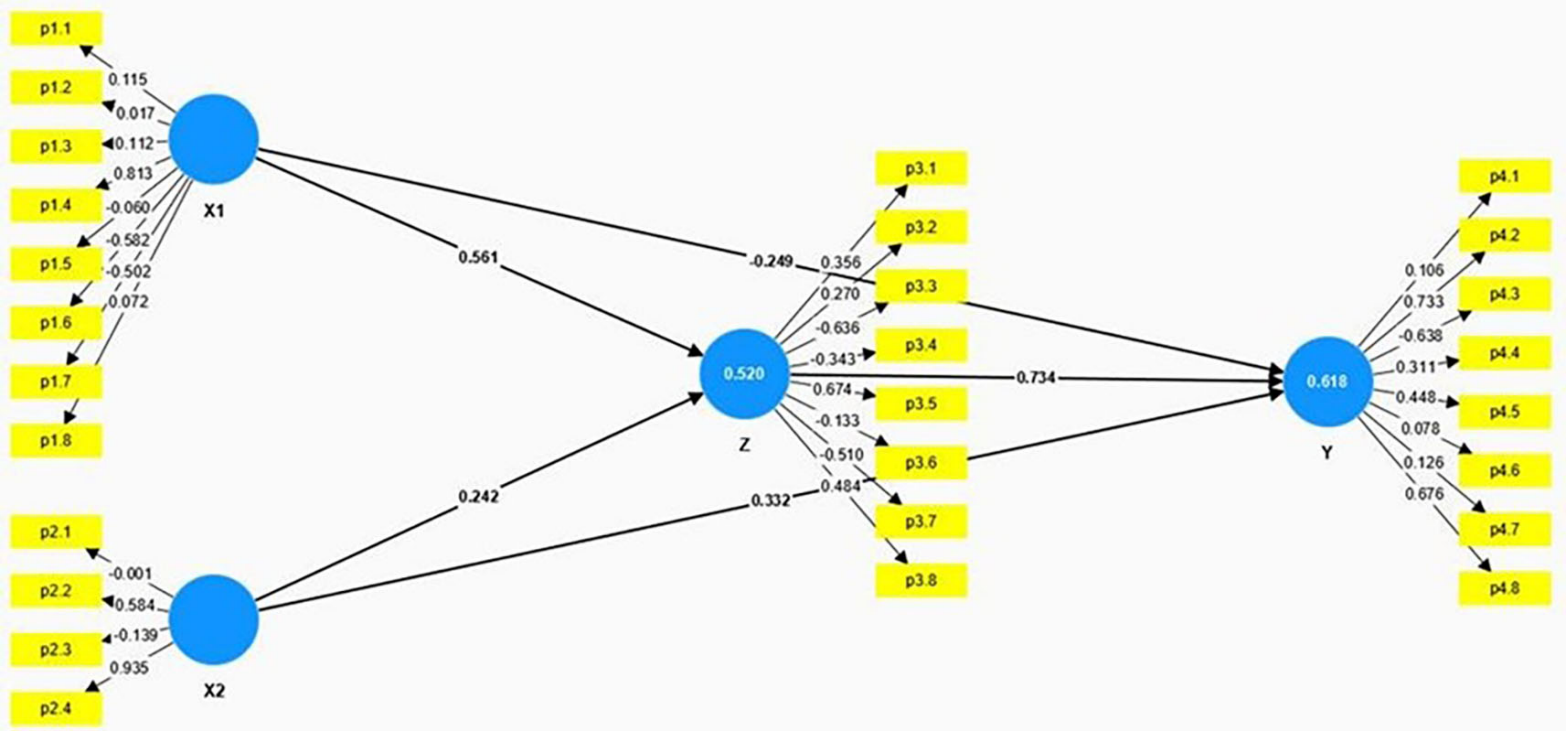
Result of hypothesis testing model.

The structural model presented in the Figure 2 and Table 3 illustrates the relationships among Anxiety (X1), Educational Level (X2), Spiritual Intelligence (Z), and Mental Health (Y). The model employs path coefficients to demonstrate the strength and direction of these relationships.

**Table 3.** Direct effect test results.

Direct effects	Path Coefficients	T-Statistics	p-values	Results
X1 ➔ Z	0.561	4.742	0.000	Significant
X2 ➔ Z	0.242	2.667	0.010	Significant
X1 ➔ Y	-0.249	3.999	0.000	Significant
X2 ➔ Y	0.332	2.597	0.013	Significant
Z ➔ Y	0.734	5.342	0.000	Significant

Anxiety (X1) has a significant positive direct effect on Spiritual Intelligence (Z), with a path coefficient of 0.561, a T-Statistic of 4.742, and a p-value of 0.000. This indicates that higher levels of anxiety are significantly associated with higher levels of spiritual intelligence. While this result may seem counterintuitive, it is possible that individuals experiencing anxiety may actively seek coping mechanisms, such as spiritual practices, to manage their distress, which may, in turn, elevate their spiritual intelligence. This could also indicate a reverse causality where individuals with heightened spiritual awareness are more sensitive to existential concerns, thereby experiencing higher anxiety levels. However, Anxiety has a significant negative direct effect on Mental Health (Y), with a path coefficient of -0.249, a T-Statistic of 3.999, and a p-value of 0.000. This suggests that higher anxiety levels are associated with poorer mental health outcomes.

Educational Level (X2) has a moderate positive effect on Spiritual Intelligence (Z), with a path coefficient of 0.242, a T-Statistic of 2.667, and a p-value of 0.010. This indicates that higher educational levels are significantly associated with higher spiritual intelligence. Educational Level also positively affects Mental Health (Y), with a path coefficient of 0.332, a T-Statistic of 2.597, and a p-value of 0.013. This shows that higher educational levels are associated with better mental health outcomes.

Spiritual Intelligence (Z) has a strong positive effect on Mental Health (Y), with a path coefficient of 0.734, a T-Statistic of 5.342, and a p-value of 0.000. This underscores the crucial role of spiritual intelligence in enhancing mental health, indicating that individuals with higher spiritual intelligence tend to have better mental health outcomes.

The model also examines the indirect effects of Anxiety (X1) and Educational Level (X2) on Mental Health (Y) through Spiritual Intelligence (Z). The indirect effect in Table 4 of Anxiety on Mental Health through Spiritual Intelligence is significant, with a calculated effect of 0.412 (0.561 * 0.734), indicating that higher levels of anxiety contribute to better mental health indirectly by enhancing spiritual intelligence. Similarly, the indirect effect of Educational Level on Mental Health through Spiritual Intelligence is also significant, with a calculated effect of 0.178 (0.242 * 0.734), suggesting that higher educational levels improve mental health indirectly by increasing spiritual intelligence. These significant indirect effects highlight the mediating role of spiritual intelligence in the relationship between anxiety, educational level, and mental health.

**Table 4.** Indirect effect test results.

Indirect effect	Path Coefficients	P-Values	Results
X1 ➔ Z ➔ Y	0.561 X 0.734 = 0.412	0.000	Significant
X2 ➔ Z ➔ Y	0.242 X 0.734 = 0.178	0.000	Significant

The summary Table 5 below presents the direct, indirect, and total effects of the variables on each other within the model:

**Table 5.** Total effect test results.

Relationship	Direct effect	Indirect effect	Total effect
X1 ➔ Z	0.561	-	0.561
X2 ➔ Z	0.242	-	0.242
X1 ➔ Y	-0.249	0.412	0.163
X2 ➔ Y	0.332	0.178	0.510
Z ➔ Y	0.734	-	0.734

The direct effect of Anxiety (X1) on Spiritual Intelligence (Z) is 0.561, while the direct effect of Educational Level (X2) on Spiritual Intelligence is 0.242. Both of these effects are significant, indicating that higher anxiety and higher educational levels lead to increased spiritual intelligence. For the relationship between Anxiety and Mental Health (Y), the direct effect is -0.249, suggesting a negative impact. However, when considering the indirect effect through Spiritual Intelligence (0.412), the total effect of Anxiety on Mental Health is 0.163, highlighting a net positive impact due to the mediation by spiritual intelligence.

Educational Level (X2) has a direct positive effect on Mental Health (0.332), and with an additional indirect effect through Spiritual Intelligence (0.178), the total effect on Mental Health is 0.510, emphasizing the substantial positive influence of education on mental health. Lastly, the direct effect of Spiritual Intelligence on Mental Health is notably strong at 0.734, reaffirming its crucial role in promoting better mental health outcomes.

In summary, the results indicate significant relationships among anxiety, educational level, spiritual intelligence, and mental health, supporting the study’s hypotheses. The positive relationship between anxiety and spiritual intelligence, while somewhat unexpected, may indicate that individuals under psychological distress seek meaning or spiritual support as a coping mechanism, indirectly enhancing their resilience. The findings reinforce the crucial role of spiritual intelligence in mental health, suggesting it as an effective focus for mental health interventions, especially in post-COVID-19 contexts.

## 5. Discussion and implication

### 5.1 The effect of anxiety on spiritual intelligence and mental health

Our findings support the hypothesis that anxiety has a significant impact on both spiritual intelligence and mental health. The positive relationship between anxiety and spiritual intelligence, while counterintuitive, suggests that individuals experiencing heightened anxiety may turn to spiritual practices or seek deeper meaning as coping mechanisms to alleviate distress. This result is consistent with the findings of Rathakrishnan et al. (2022), who proposed that anxiety might prompt individuals to enhance their spiritual intelligence to cope with emotional pain. Given that most of the sample consists of younger and female participants, this relationship could reflect coping preferences commonly observed within these demographics, where spiritual and emotional support are often sought in response to psychological distress. Additionally, it is worth considering the possibility of reverse causality, where heightened spiritual awareness could lead to increased sensitivity to existential concerns, thereby potentially elevating anxiety levels. Further longitudinal studies would be needed to clarify the direction of this relationship.

However, anxiety’s negative impact on mental health is evident, emphasizing the need for interventions that address both direct and indirect effects of anxiety. Spiritual intelligence appears to buffer some of these negative impacts by promoting emotional resilience and a sense of purpose, thereby helping to reduce the detrimental effects of anxiety on mental well-being. This suggests that integrating spiritual development practices in mental health treatments for anxiety could offer protective effects. Practices like mindfulness, meditation, and spiritual counseling could support individuals in finding purpose, thus potentially reducing the adverse effects of anxiety on mental health. This approach aligns with the buffering theory of Rathakrishnan et al. (2022), which proposes that spiritual intelligence enhances emotional resilience, mitigating the harmful effects of anxiety.

### 5.2 The effect of educational level on spiritual intelligence and mental health

Spiritual intelligence and mental health are strongly influenced by educational level. Increased cognitive and emotional growth is probably the reason why higher educational attainment is associated with increased spiritual intelligence. This confirms Putra’s (2016) findings that learning promotes understanding and spiritual growth. Additionally, studies by Taple et al. (2022), Raghupathi and Raghupathi (2020), and Namira and Yuliawati (2021) that highlight the advantages of higher education in providing access to better employment opportunities, financial security, and healthcare, tend to support the idea that people with higher educational levels also tend to have better mental health outcomes. Education thus serves as a foundation for developing spiritual intelligence, equipping individuals with the knowledge and skills needed to cultivate spiritual awareness and practices, which positively impact mental health.

The significant influence of education on spiritual intelligence and mental health highlights the importance of educational policies that promote mental well-being. Incorporating curricula that support spiritual as well as cognitive and emotional development could be particularly beneficial for younger populations, as seen in this study. Programs that foster empathy, ethical reasoning, and critical thinking could enhance students’ spiritual intelligence and, in turn, their mental health. This approach supports findings from Taple et al. (2022), Raghupathi and Raghupathi (2020), and Namira and Yuliawati (2021), which show how education can enhance mental health outcomes by giving people better access to resources and support systems.

### 5.3 The effect of spiritual intelligence on mental health

Spiritual intelligence plays a critical role in promoting positive mental health outcomes. Individuals with high levels of spiritual intelligence are generally better equipped to manage stress and anxiety, which contributes to improved mental health. This result is consistent with studies by Wahyuni and Bariyyah (2019) and Rathakrishnan et al. (2022), which indicate that spiritual intelligence supports people in exhibiting empathy and compassion, finding meaning and purpose in life, and preserving inner peace—all of which are factors that positively impact mental health. Given that the sample primarily consists of younger and female participants, this strong relationship between spiritual intelligence and mental health may align with the supportive and meaning-seeking characteristics often observed in these groups. However, it is essential to note Furqani (2021) findings, which indicated variability in this relationship among different groups, suggesting that the impact of spiritual intelligence on mental health may depend on cultural or demographic factors.

The substantial positive correlation between spiritual intelligence and mental health bears important implications for interventions aimed at promoting mental health. Policymakers and mental health professionals could consider incorporating spiritual intelligence development into their programs. For instance, interventions aimed at helping individuals find purpose, maintain inner calm, and develop empathy could enable them to better cope with stress and anxiety. This is consistent with the favorable results reported by Wahyuni and Bariyyah (2019) and Rathakrishnan et al. (2022), which suggests that spiritual intelligence can be an effective means of fostering mental wellness. Notwithstanding the inconclusive results reported by Furqani (2021), it is imperative to customize these initiatives to particular cultural and demographic settings in order to guarantee their efficacy.

## 6. Conclusion

Understanding the mediating role of spiritual intelligence in the relationships between anxiety, education level, and mental health has practical implications for designing comprehensive mental health interventions. Programs that simultaneously address anxiety management, educational attainment, and spiritual development are likely to be more effective in promoting mental well-being. This holistic approach allows individuals to benefit from both direct and indirect pathways identified in this study. To support integrated mental health systems, policies should encourage interdisciplinary collaboration among educators, mental health practitioners, and spiritual counselors.

However, this study has several limitations. First, the cross-sectional design limits causal inferences between variables, and future research could address this through longitudinal studies to observe changes over time. Second, the specific focus on post-COVID-19 patients in Indonesia may limit the generalizability of these findings to other populations or settings. Expanding the sample to include more diverse groups could strengthen the applicability of the results. Lastly, while this study emphasizes the role of spiritual intelligence, it does not fully delve into how its distinct dimensions individually contribute to mental health outcomes, which could be explored further in future research.

Despite these limitations, these findings offer insightful information for both scholarly study and real-world interventions meant to improve mental health. Putting a strong emphasis on the growth of spiritual intelligence may be a calculated move to lessen the negative effects of anxiety and maximize the benefits of education. To validate and build on these findings, future studies should investigate these correlations in a variety of demographics and circumstances.

## Ethics and consent

All procedures involving human participants in this study were conducted in accordance with the institutional ethical standards of Institut Ilmu Kesehatan Strada Indonesia, which received approval under number 000986/EC/KEPK/I/03/2024 on March 7, 2024. The health and well-being of the participants were prioritized throughout the study, in line with the commitment to ensuring their safety and ethical treatment.

Informed consent was obtained from all participants. Written consent was provided by each participant, who received comprehensive information about the study’s purpose, procedures, potential risks, and benefits. Participants voluntarily agreed to participate in the study based on this information.

## Data Availability

The data presented in this study are available on request from the corresponding author due to confidentiality agreements with the participants involved in the research. Our data statement is complete and adheres to the journal’s guidelines. Access to the data is restricted to protect participant privacy. Researchers wishing to access the data must submit a formal request to the corresponding author, detailing the purpose of their research, the specific data needed, intended use, and measures for ensuring data security and participant confidentiality. Requests will be evaluated on a case-by-case basis, and access will be granted under specific conditions approved by our Institutional Review Board (IRB). For further inquiries, please contact anisansyori@itsk-soepraoen.ac.id. Figshare: Questionnaire for “Examining the Effects of Anxiety and Education Level on Mental Health: The Role of Spiritual Intelligence as an Intervening Variable in Post COVID-19 Patients in Indonesia”, DOI: https://doi.org/10.6084/m9.figshare.26422345.v1 ( Ansyori A, et al., 2024a). STROBE checklist for “Examining the Effects of Anxiety and Education Level on Mental Health: The Role of Spiritual Intelligence as an Intervening Variable in Post COVID-19 Patients in Indonesia”, DOI: https://doi.org/10.6084/m9.figshare.26422324.v1 ( Ansyori A, et al., 2024b). Data are available under the terms of the Creative Commons Attribution 4.0 International license (CC-BY 4.0).
