# Supplementary material for: Examining the effects of anxiety and education level on mental health: The role of spiritual intelligence as an intervening variable in post COVID-19 patients in Indonesia
Source: F1000Res. 2025 Feb 18;13:916. Originally published 2024 Aug 12. [Version 4] doi: 10.12688/f1000research.154599.4 (PMC11826076; doi:10.12688/f1000research.154599.4)
Supplement: Peer review history

# Reviewer report: Reviewer response for version 4

**DOI:** 10.5256/f1000research.178246.r339441

**Published:** 2025-05-12

**Authors:** M Arli Rusandi

**Affiliations:** 1 Department of Guidance and Counseling, Universitas Riau, Pekanbaru, Riau, Indonesia

No more comments from me, because the authors have fixed it.

Is the work clearly and accurately presented and does it cite the current literature?

Yes

If applicable, is the statistical analysis and its interpretation appropriate?

Yes

Are all the source data underlying the results available to ensure full reproducibility?

Partly

Is the study design appropriate and is the work technically sound?

Yes

Are the conclusions drawn adequately supported by the results?

Yes

Are sufficient details of methods and analysis provided to allow replication by others?

Partly

Reviewer Expertise:

education, counseling, career development, digital health, happenstance lerarning

I confirm that I have read this submission and believe that I have an appropriate level of expertise to confirm that it is of an acceptable scientific standard.

# Reviewer report: Reviewer response for version 4

**DOI:** 10.5256/f1000research.178246.r367296

**Published:** 2025-02-19

**Authors:** M Arli Rusandi

**Affiliations:** 1 Department of Guidance and Counseling, Universitas Riau, Pekanbaru, Riau, Indonesia

The authors have made substantial and well-executed revisions that directly address my concerns. The study now presents a well-structured and compelling analysis of how anxiety, education level, and spiritual intelligence shape mental health outcomes among post-COVID-19 patients in Indonesia. The introduction clearly states the research objectives and highlights the study’s relevance within the Indonesian post-pandemic context. The refined literature review eliminates redundancy while maintaining a strong theoretical foundation and identifying the research gap. Additionally, the improved methodological transparency enhances the study’s credibility. The authors acknowledge potential biases from the purposive sampling method and explain how they adapted and validated measurement tools to fit the Indonesian context.

 One of the most commendable aspects of this revision is the thorough explanation of the counterintuitive finding that anxiety positively influences spiritual intelligence. The discussion now explores theoretical interpretations of this unexpected result, considering factors such as reverse causality and cultural influences. Furthermore, the revised manuscript explicitly outlines ethical considerations and data availability, detailing a structured procedure for requesting access to the study’s data while ensuring participant confidentiality. The discussion also incorporates practical recommendations, offering concrete suggestions on integrating spiritual intelligence into mental health interventions.

 The authors have also effectively addressed minor suggestions, such as refining keywords, improving readability, and strengthening the concluding statement on policy implications. Overall, the manuscript is now well-prepared for indexing. This study makes a valuable contribution to mental health research, particularly in post-pandemic settings, by providing important insights into the role of spiritual intelligence as a mediating factor in mental health outcomes. Its clarity, structure, and depth make it an engaging and informative read. No further revisions are necessary, and I recommend the manuscript for acceptance in its current form.

Is the work clearly and accurately presented and does it cite the current literature?

Yes

If applicable, is the statistical analysis and its interpretation appropriate?

Yes

Are all the source data underlying the results available to ensure full reproducibility?

Partly

Is the study design appropriate and is the work technically sound?

Yes

Are the conclusions drawn adequately supported by the results?

Yes

Are sufficient details of methods and analysis provided to allow replication by others?

Partly

Reviewer Expertise:

education, counseling, career development, digital health, happenstance lerarning

I confirm that I have read this submission and believe that I have an appropriate level of expertise to confirm that it is of an acceptable scientific standard.

# Reviewer report: Reviewer response for version 3

**DOI:** 10.5256/f1000research.176909.r365588

**Published:** 2025-02-13

**Authors:** Nurshahira Ibrahim

**Affiliations:** 1 Universiti Teknologi MARA, JENGKA, Pahang, Malaysia

1. Is the study design appropriate and is the work technically sound? Answer: Partly

 - The cross-sectional design is appropriate for the research objectives, and the use of Structural Equation Modeling (SEM) is suitable for analyzing complex relationships between variables. However, there are some concerns:

 1. The cross-sectional nature limits causal inferences, which should be more explicitly acknowledged as a limitation.

 2. The adaptation and validation process of the measurement scales for the Indonesian context could be more thoroughly explained to ensure cultural relevance and validity.

 2. Are sufficient details of methods and analysis provided to allow replication by others?

 Answer: Partly

 - While the methodology section is comprehensive, there are areas that could be improved to enhance replicability:

 1. More details on the adaptation and validation of measurement scales for the Indonesian context would be beneficial.

 2. The specific procedures for data collection and handling could be elaborated.

 3. A more detailed description of the SEM analysis process, including software used and model fit indices, would aid replication.

 3. Are all the source data underlying the results available to ensure full reproducibility?

 Answer: Partly

 - The authors mention that data are available on request due to confidentiality agreements. While this protects participant privacy, it may limit full reproducibility. To address this:

 1. Consider providing a more detailed data availability statement, including the process for requesting access to the data.

 2. If possible, provide anonymized datasets or summary statistics that do not compromise participant confidentiality.

 Recommendations:

 1. Strengthen the discussion of limitations, particularly regarding the cross-sectional design and its implications for causal inferences.

 2. Provide more detailed information on the adaptation and validation of measurement scales for the Indonesian context to ensure cultural relevance and validity.

 3. Elaborate on the data collection procedures and SEM analysis process to enhance replicability.

 4. Consider offering more concrete recommendations for implementing spiritual intelligence development in mental health interventions and educational programs.

 5. Address the demographic skew towards younger, female participants and discuss how this might affect the generalizability of findings.

 6. Explore alternative explanations for the positive relationship between anxiety and spiritual intelligence, including the possibility of reverse causality.

 7. Enhance the data availability statement to provide clearer guidelines for accessing the data while maintaining participant confidentiality.

 8. Consider adding a brief discussion of ethical considerations in the methodology section, in addition to the existing mention in the conclusion.

Is the work clearly and accurately presented and does it cite the current literature?

Yes

If applicable, is the statistical analysis and its interpretation appropriate?

Yes

Are all the source data underlying the results available to ensure full reproducibility?

Partly

Is the study design appropriate and is the work technically sound?

Partly

Are the conclusions drawn adequately supported by the results?

Yes

Are sufficient details of methods and analysis provided to allow replication by others?

Partly

Reviewer Expertise:

My expertise is in spiritual intelligence (psychology context).

I confirm that I have read this submission and believe that I have an appropriate level of expertise to confirm that it is of an acceptable scientific standard, however I have significant reservations, as outlined above.

# Reviewer report: Reviewer response for version 3

**DOI:** 10.5256/f1000research.176909.r359983

**Published:** 2025-02-07

**Authors:** M Arli Rusandi

**Affiliations:** 1 Department of Guidance and Counseling, Universitas Riau, Pekanbaru, Riau, Indonesia

**Summary of Revision Evaluation – Round 2.**

- Significant improvements have been made throughout the manuscript.
- The **sections of the Introduction, Methods, Results, Discussion, and Conclusion** have been appropriately revised according to the previous suggestions.
- No significant inconsistencies or notable methodological errors remain after this revision.
  --------------------------
- **One minor issue remains**: The keywords have not been updated as previously agreed.

**Final Suggestion**:

 If the keywords are revised, the manuscript can be considered well-revised and ready for the next stage.

Is the work clearly and accurately presented and does it cite the current literature?

Yes

If applicable, is the statistical analysis and its interpretation appropriate?

Yes

Are all the source data underlying the results available to ensure full reproducibility?

Partly

Is the study design appropriate and is the work technically sound?

Yes

Are the conclusions drawn adequately supported by the results?

Yes

Are sufficient details of methods and analysis provided to allow replication by others?

Partly

Reviewer Expertise:

education, counseling, career development, digital health, happenstance lerarning

I confirm that I have read this submission and believe that I have an appropriate level of expertise to confirm that it is of an acceptable scientific standard, however I have significant reservations, as outlined above.

# Response

**Published:** 2025-02-13

**Authors:** Anis Ansyori

**Affiliations:** Public Health, Institut Ilmu Kesehatan Strada Indonesia, Kediri, East Java, Indonesia

Thank you for your valuable feedback and for acknowledging the improvements made throughout the manuscript. I have now updated the keywords as requested. Please find the revised version reflecting the necessary changes.

# Reviewer report: Reviewer response for version 3

**DOI:** 10.5256/f1000research.176909.r359984

**Published:** 2025-01-28

**Authors:** Yuni Asri

**Affiliations:** 1 Department of Nursing, Faculty of Health and Science, Institut Teknologi, Sains dan Kesehatan RS dr Soepraoen Kesdam V/Brawijaya, Malang, Indonesia

Thank you for addressing the feedback thoroughly and providing detailed responses to the suggested revisions. I appreciate the effort and thoughtfulness reflected in your revisions.

 Overall, these revisions have significantly improved the clarity, transparency, and academic quality of the manuscript. I am satisfied with the changes made and believe they have strengthened the study.

 Thank you for your thoughtful responses and the improvements made to the manuscript.

Is the work clearly and accurately presented and does it cite the current literature?

Yes

If applicable, is the statistical analysis and its interpretation appropriate?

Partly

Are all the source data underlying the results available to ensure full reproducibility?

Partly

Is the study design appropriate and is the work technically sound?

Partly

Are the conclusions drawn adequately supported by the results?

Yes

Are sufficient details of methods and analysis provided to allow replication by others?

Yes

Reviewer Expertise:

Based on my expertise in Public Health, there are potential research areas that focus on:Mental Health and Psychosocial Support, Health Inequities, Global Health and health policy

I confirm that I have read this submission and believe that I have an appropriate level of expertise to confirm that it is of an acceptable scientific standard.

# Reviewer report: Reviewer response for version 2

**DOI:** 10.5256/f1000research.174458.r341019

**Published:** 2024-12-06

**Authors:** Yuni Asri

**Affiliations:** 1 Department of Nursing, Faculty of Health and Science, Institut Teknologi, Sains dan Kesehatan RS dr Soepraoen Kesdam V/Brawijaya, Malang, Indonesia

1. **Study Design and Academic Merit. **Structural Equation Modeling (SEM) is appropriate for analyzing complex interrelations among variables. The incorporation of spiritual intelligence as a mediator is innovative and contributes to the academic merits of this study. While the cross-sectional design suits the study objectives, it limits the causal inferences. This should be highlighted more prominently as a limitation of this study. Discuss how the adapted scales were validated for cultural relevance in the Indonesian context.
2. **Statistical Analysis and Interpretation. **This manuscript reports a robust statistical analysis with clearly explained path coefficients and mediation effects. The positive relationship between anxiety and spiritual intelligence, while counterintuitive, can be explained as follows. However, additional alternative interpretations and supporting evidence could strengthen this argument. To address potential reverse causality between spiritual intelligence and anxiety more explicitly
3. **Reproducibility of Results**. The data availability statement mentions restrictions owing to confidentiality. To enhance reproducibility, we considered providing anonymized datasets or detailed procedures for qualified researchers to access the data. When the scales and tools are cited, their full descriptions or references should be included to ensure reproducibility

Is the work clearly and accurately presented and does it cite the current literature?

Yes

If applicable, is the statistical analysis and its interpretation appropriate?

Partly

Are all the source data underlying the results available to ensure full reproducibility?

Partly

Is the study design appropriate and is the work technically sound?

Partly

Are the conclusions drawn adequately supported by the results?

Yes

Are sufficient details of methods and analysis provided to allow replication by others?

Yes

Reviewer Expertise:

Based on my expertise in Public Health, there are potential research areas that focus on:Mental Health and Psychosocial Support, Health Inequities, Global Health and health policy

I confirm that I have read this submission and believe that I have an appropriate level of expertise to confirm that it is of an acceptable scientific standard, however I have significant reservations, as outlined above.

# Response

**Published:** 2025-01-10

**Authors:** Anis Ansyori

**Affiliations:** Public Health, Institut Ilmu Kesehatan Strada Indonesia, Kediri, East Java, Indonesia

Dear Reviewer,

 Thank you for your valuable feedback. We have carefully revised the manuscript based on your suggestions. Below are the key updates:

**1. Study Design and Academic Merit**

 We have emphasized the limitation of SEM in establishing causality, highlighting that the cross-sectional design restricts causal inferences. To address this, we suggest future longitudinal studies for better understanding. Additionally, we have expanded the validation process of adapted scales, detailing content validation, construct validity assessment, and reliability testing to ensure cultural relevance in the Indonesian context.

**2. Statistical Analysis and Interpretation**

 We have added alternative interpretations and supporting evidence for the relationship between anxiety and spiritual intelligence. Some studies (e.g., Jamshidi et al., 2017) support a positive relationship, while others (Khoshdeli et al., 2020; Safavi et al., 2019; Mohammadipour et al., 2021) suggest a negative effect, where spiritual intelligence helps reduce anxiety. We have acknowledged these differences and also addressed reverse causality, proposing that higher spiritual awareness may increase existential concerns, potentially elevating anxiety.

**3. Reproducibility of Results**

 Due to confidentiality agreements, we cannot provide an anonymized dataset. However, we have updated the data availability statement, outlining a formal request process for researchers. Requests must include name, institution, research purpose, specific data needed, and ethical clearance. Each request will be reviewed on a case-by-case basis under Institutional Review Board (IRB) approval. Additionally, we have ensured complete references and descriptions of measurement tools to enhance reproducibility.

 These revisions improve the clarity, validity, and transparency of the study. We appreciate your insightful feedback, which has strengthened our manuscript.

# Reviewer report: Reviewer response for version 1

**DOI:** 10.5256/f1000research.169647.r314076

**Published:** 2024-09-12

**Authors:** M Arli Rusandi

**Affiliations:** 1 Department of Guidance and Counseling, Universitas Riau, Pekanbaru, Riau, Indonesia

**Summary of the Article:**

 The article investigates the relationships among anxiety, education level, spiritual intelligence, and mental health in post-COVID-19 patients in Indonesia. Utilizing a cross-sectional design and Structural Equation Modeling (SEM), the study analyzes data collected from 390 participants. The findings reveal that anxiety significantly influences both spiritual intelligence and mental health, with spiritual intelligence serving as a mediating factor. Moreover, the study demonstrates that higher educational attainment is positively correlated with enhanced spiritual intelligence and better mental health outcomes. The article concludes by highlighting the significance of incorporating spiritual development practices into mental health interventions and educational programs to enhance resilience and overall well-being in the post-pandemic context.

**Evaluation Based on Questionnaire:**

 3. Are sufficient details of methods and analysis provided to allow replication by others?

**Answer:** Partly, Although the methods section is comprehensive and notes the use of established measurement scales, the manuscript would benefit from further elaboration on the validation or adaptation of these scales for the Indonesian context. Providing these additional details would enhance the study's replicability, particularly for researchers operating in different cultural settings.

**5. Are all the source data underlying the results available to ensure full reproducibility?**

**Answer:** Partly, the manuscript discusses the availability of data, with some restrictions due to participant confidentiality. While the data availability statement is clear, these restrictions may limit the ability to fully reproduce the study's findings. The authors might consider offering anonymized data or providing further details on how the data can be accessed by qualified researchers.

**Reviewer Comments to Author**

 To ensure comprehensiveness, I will organize my comments according to the manuscript's structure.

**Introduction**

**Major Comments:**

 1. The introduction provides a solid foundation by addressing the impact of the COVID-19 pandemic on mental health, particularly within the Indonesian context. However, the research aim or question could be articulated more explicitly earlier in the introduction. This would help orient the reader and clarify the study's purpose from the outset.

 2. The rationale for exploring the role of spiritual intelligence as a mediator between anxiety, education, and mental health is well-founded and relevant. However, consider summarizing the existing literature more concisely to avoid redundancy and maintain a sharper focus on the specific research gap your study aims to address.

**Minor Comments:**

 1. Ensure consistent use of terminology when discussing mental health. The text occasionally shifts between general references to mental health and specific conditions such as anxiety and depression. It may be beneficial to clarify early in the manuscript whether the study focuses broadly on mental health or specifically on conditions like anxiety and depression.

 2. The transitions between paragraphs could be smoother, especially when shifting from the general discussion of the pandemic’s impact to the specific focus on spiritual intelligence. Consider adding transitional sentences to enhance the narrative flow.

**Methodology**

**Major Comments:**

 1. The use of purposive non-probability sampling is appropriate given the specific population targeted by the study. However, this approach introduces potential biases that should be acknowledged. It would be beneficial to discuss how these biases might impact the generalizability of the findings and whether any measures were taken to mitigate them.

 2. The measurement tools selected for the study appear well-chosen and likely valid. However, it would be advantageous to provide additional details regarding how these tools were adapted or validated for the Indonesian context, particularly considering potential cultural differences in the interpretation of survey items.

**Minor Comments:**

 1. The inclusion and exclusion criteria are clearly articulated and well-defined. However, the exclusion of patients with chronic illnesses could limit the generalizability of the findings. A brief discussion of this limitation would enhance the transparency of the study.

 Ethical Considerations:

 2. While the methodology section is comprehensive, including a brief mention of the ethical considerations or approval process in this section, in addition to the detailed explanation in the conclusion, would further underscore the study's ethical integrity.

**Results**

**Major Comments:**

 1. The results are presented with clarity, and the significant findings effectively support your hypotheses. However, the positive relationship observed between anxiety and spiritual intelligence is somewhat counterintuitive. It would be beneficial to explore alternative explanations for this finding or to consider the possibility of reverse causality, either within the results section or in greater detail in the discussion.

 2. The significant indirect effects mediated by spiritual intelligence are a crucial aspect of the study's findings. It is important to ensure that these effects are clearly interpreted, particularly in terms of their practical implications for mental health interventions.

**Minor Comments:**

 1. While the demographic data provided is comprehensive, the skew towards younger, female participants should be addressed. Consider discussing how this demographic imbalance might affect the generalizability of your findings to a broader population.

 2. The results section could benefit from a brief summary at its conclusion, linking the findings back to the original research questions or hypotheses. This would reinforce the narrative and help ensure that the reader fully grasps the significance of the results.

**Discussion and Conclusions**

**Major Comments:**

 1. The discussion successfully connects your findings to the existing literature, especially in emphasizing the role of spiritual intelligence. However, the counterintuitive result that anxiety positively influences spiritual intelligence warrants a more thorough exploration. Consider addressing any conflicting literature or offering theoretical explanations to better contextualize this finding.

 2. The discussion of limitations is appropriately transparent, particularly with regard to the cross-sectional design and the specificity of the population sample. Expanding on how these limitations could be addressed in future research—such as through longitudinal studies or more diverse sampling—would enhance the robustness of your recommendations.

**Minor Comments:**

 1. The discussion of integrating spiritual intelligence into mental health interventions is well-argued. However, providing additional concrete examples or recommendations for implementation in educational or clinical settings could strengthen this section.

 2. The conclusion effectively summarizes the study’s contributions and acknowledges its limitations. Including a final sentence that emphasizes the broader impact of your findings on mental health policy or practice would create a stronger and more impactful closing statement.

 Abstract

**Major Comments:**

 The abstract is well-organized and effectively summarizes the study. However, including a brief mention of the study’s limitations or the need for further research would provide a more comprehensive overview. Additionally, specifying the statistical methods used, such as Structural Equation Modeling (SEM), could add depth and clarity.

**Minor Comments:**

 Keywords:

 The keywords are appropriate, but you might consider adding terms like "post-pandemic" or "cross-sectional study" to improve searchability and relevance.

Is the work clearly and accurately presented and does it cite the current literature?

Yes

If applicable, is the statistical analysis and its interpretation appropriate?

Yes

Are all the source data underlying the results available to ensure full reproducibility?

Partly

Is the study design appropriate and is the work technically sound?

Yes

Are the conclusions drawn adequately supported by the results?

Yes

Are sufficient details of methods and analysis provided to allow replication by others?

Partly

Reviewer Expertise:

My areas of expertise include Education, Counseling, Digital Health, Information Technology Education, Higher Education, and Artificial Intelligence in Education.

I confirm that I have read this submission and believe that I have an appropriate level of expertise to confirm that it is of an acceptable scientific standard, however I have significant reservations, as outlined above.

# Response

**Published:** 2024-11-05

**Authors:** Anis Ansyori

**Affiliations:** Public Health, Institut Ilmu Kesehatan Strada Indonesia, Kediri, East Java, Indonesia

Thank you for your thorough review and valuable feedback on our manuscript. We appreciate the time and expertise you dedicated to enhancing the quality of our study. Below, we address each of your comments and outline the corresponding revisions we have made to the manuscript.

**Introduction**

1. **Research Aim/Question**: We have revised the introduction to state the research aim more explicitly at the beginning to improve clarity and orientation for readers.
2. **Concise Literature Summary**: We have streamlined the literature review to maintain focus on the specific research gap related to the role of spiritual intelligence, reducing redundancy as suggested.
3. **Terminology Consistency**: We have ensured consistent terminology when discussing mental health, specifying whether we refer to mental health in general or specific conditions such as anxiety and depression.
4. **Paragraph Transitions**: Transitional sentences have been added to create a smoother flow, particularly when moving from the pandemic’s impact to the focus on spiritual intelligence.

**Methodology**

1. **Sampling and Potential Biases**: We have acknowledged potential biases from using purposive non-probability sampling and discussed how these biases might impact generalizability.
2. **Adaptation/Validation of Tools**: Additional details have been added regarding the adaptation and validation of measurement tools for the Indonesian context, with consideration of cultural factors in interpreting survey items.
3. **Inclusion and Exclusion Criteria**: We have discussed how excluding patients with chronic illnesses may limit the generalizability of findings, as recommended.
4. **Ethical Considerations**: We have briefly mentioned ethical considerations, including the approval process, in the methodology section to emphasize the study’s ethical integrity.

**Results**

1. **Anxiety and Spiritual Intelligence**: In response to the counterintuitive positive relationship between anxiety and spiritual intelligence, we have expanded the discussion to include possible explanations, such as coping mechanisms and reverse causality, and referenced relevant literature to contextualize this finding.
2. **Interpretation of Indirect Effects**: We have clarified the interpretation of significant indirect effects mediated by spiritual intelligence, particularly regarding their practical implications for mental health interventions.
3. **Demographic Data**: We have addressed the demographic skew towards younger, female participants and discussed how this may affect the generalizability of our findings.
4. **Summary of Results**: A brief summary has been added at the end of the results section to link the findings back to the research questions, reinforcing the significance of the results.

**Discussion and Conclusions**

1. **Counterintuitive Finding on Anxiety and Spiritual Intelligence**: We have provided a more detailed exploration of the unexpected positive relationship between anxiety and spiritual intelligence, referencing possible explanations and conflicting literature.
2. **Discussion of Limitations and Future Research**: We have expanded on how limitations, such as the cross-sectional design and specific sample population, could be addressed in future research, including suggestions for longitudinal studies and broader sampling.
3. **Concrete Examples for Interventions**: In the discussion on integrating spiritual intelligence into mental health interventions, we have added practical recommendations for implementation in educational and clinical settings.
4. **Final Impact Statement**: We have strengthened the conclusion with a closing statement that highlights the broader impact of our findings on mental health policy and practice.

**Abstract and Keywords**

1. **Limitations and Future Research**: We have updated the abstract to briefly mention the study’s limitations and the need for further research.
2. **Statistical Methods**: We have included the statistical method used (Structural Equation Modeling) to add clarity.
3. **Keywords**: Terms like “post-pandemic” and “cross-sectional study” have been added to improve searchability and relevance.

Once again, we thank you for your constructive feedback, which has greatly helped us refine and strengthen our manuscript. We hope that our responses and revisions meet your expectations and enhance the scientific quality and clarity of our study.

 Sincerely,

 Authors
